# Tissue-Specific Remodeling and Load Partitioning in Maxillary Expansion: An Evidence-Informed Three-Compartment Mechanobiological Framework

**DOI:** 10.3390/bioengineering13091067

**Published:** 2026-09-14

**Authors:** Grzegorz Hajduk, Paulina Kuc, Natalia Kuc, Stanisław Hajduk, Michał Sarul, Anna Ewa Kuc

**Affiliations:** 1 Chair and Department of Oral Surgery, Medical University of Lublin, Witolda Chodźki 6, 20-093 Lublin, Poland; 2 Faculty of Medicine, Medical University in Bialystok, ul. Kilińskiego 1, 15-089 Bialystok, Poland; 3 Faculty of Dentistry, Medical University of Lublin, Witolda Chodźki 6, 20-093 Lublin, Poland; 4 Department of Integrated Dentistry, Wroclaw Medical University, Krakowska 26, 50-425 Wroclaw, Poland; 5 Department of Dentofacial Orthopedics and Orthodontics, Wroclaw Medical University, 50-425 Wroclaw, Poland; annaewakuc@wp.pl

**Keywords:** maxillary expansion, midpalatal suture, circummaxillary sutures, alveolar bone, periodontal ligament, mechanobiology, load partitioning, tissue-specific remodeling, skeletal anchorage

## Abstract

Maxillary expansion combines sutural skeletal displacement with variable alveolar, dental, and periodontal responses. Because these effects are often reported as a single transverse outcome, their biological contributions are difficult to separate. We reviewed clinical, histological, and experimental evidence on midpalatal and circummaxillary sutural remodeling, alveolar deformation, anchorage-related periodontal loading, and used this evidence to formulate a three-compartment model. The sutural skeletal domain is the intended orthopedic target; the alveolar and dentoalveolar domain describes adaptive load transfer; and the anchorage-related periodontal domain captures non-target tissue responses. These labels describe treatment roles and do not imply a fixed sequence or biological independence. Experimental studies report overlapping mechanosensing, cellular recruitment, osteoclast-related margin turnover, immune and vascular activity, and osteogenesis within expanded sutures. Direct longitudinal molecular data in humans remain scarce, and the order of these events is unresolved. Clinical imaging also shows that comparable transverse corrections can contain different proportions of sutural opening, alveolar bending, dental tipping, and buccal cortical change. The model distinguishes skeletal correction from accompanying dentoalveolar and periodontal effects and yields testable predictions for multimodal imaging, tissue-specific biological measurements, histology, and patient-specific computational modeling. We present it as a provisional analytic model, not as a validated biological classification or clinical prediction rule.

## 1. Introduction

Maxillary expansion is a mechanically driven orthopedic intervention used to correct transverse maxillary deficiency through tooth-borne, tooth–bone-borne, bone-anchored, or surgically assisted systems. Although these approaches can produce clinically meaningful increases in maxillary and dental arch width, the tissue-level response is not uniform. Three-dimensional studies demonstrate variable proportions of midpalatal sutural opening, basal maxillary displacement, alveolar bending, dental tipping, and buccal cortical change, even among patients treated with broadly comparable expansion protocols [1,2,3,4,5,6].

These observations indicate that clinically measured transverse widening is a composite outcome rather than a single biological event. Intermolar or interpremolar width increase alone does not identify the relative contribution of sutural separation, circummaxillary skeletal displacement, alveolar deformation, or dental movement. In some patients, the correction contains a substantial skeletal component, whereas in others, a larger proportion is achieved through dentoalveolar compensation. Recent studies have also demonstrated heterogeneous opening patterns after miniscrew-assisted expansion, including differences in expansion efficiency, anterior–posterior parallelism, and the contribution of surrounding sutural resistance [7,8].

At the sutural level, available histological and experimental evidence indicates that expansion is accompanied by active tissue turnover rather than passive gap formation alone. Studies have reported osteoclast-related signaling at sutural margins, angiogenic mediator release, participation of neutrophils and mechanically induced macrophage phenotypes, mechanosensitive Piezo1 signaling, and tension-responsive osteogenic programs in suture stem or progenitor cells [9,10,11,12,13,14].

These findings support the existence of a biologically active sutural response, but they do not establish a single obligatory molecular cascade in humans. Most mechanistic evidence has been obtained from animal or in vitro models, and the temporal and causal relationships among direct mechanosensing, immune-cell recruitment, vascular adaptation, osteoclast-related turnover, and osteogenesis remain incompletely defined. Human evidence is strongest for structural opening, variation in skeletal expansion pattern, dentoalveolar effects, and subsequent radiographic restoration of mineralized tissue, whereas direct longitudinal sampling of the human midpalatal suture is rarely feasible [15,16].

Alveolar bone, teeth, and periodontal tissues also respond directly to expansion-related loading. Clinical imaging studies have demonstrated alveolar inclination changes, dental tipping, alterations in buccal cortical thickness, and variable periodontal effects, with their distribution influenced by anchorage configuration, patient anatomy, skeletal maturity, and appliance–bone coupling [1,2,17,18]. Gingival crevicular fluid studies performed during rapid maxillary expansion have additionally demonstrated changes in inflammatory mediators around anchorage teeth. Such measurements characterize a local periodontal response but should not be interpreted as direct biomarkers of sutural remodeling [19].

Most studies examine one tissue domain at a time. Sutural studies focus on opening, cellular turnover, vascularization, and bone formation. Clinical imaging studies quantify skeletal, alveolar, and dental effects. Periodontal studies assess anchorage-related inflammation and tissue morbidity. Few studies explain how these responses combine within the same patient or why similar protocols produce different proportions of skeletal correction and dentoalveolar burden.

This article reviews the evidence for the sutural, alveolar and dentoalveolar, and anchorage-related periodontal responses and integrates them into a three-compartment model. Within this framework, the midpalatal and circummaxillary sutural system constitutes the sutural skeletal domain and the principal intended orthopedic target; alveolar bone and the dentoalveolar complex constitute an adaptive load-transfer domain; and the anchorage-related periodontal domain constitutes a non-target tissue-response domain. The terms principal, adaptive, and non-target describe the intended role of each domain in treatment. They do not imply that one tissue always responds first, that the domains are biologically isolated, or that sutural remodeling necessarily dominates the observed clinical result in every patient.

### Scope and Evidentiary Approach

This article is a narrative article rather than a systematic review. The synthesis prioritizes studies that directly evaluate midpalatal or circummaxillary sutural response, three-dimensional clinical studies that distinguish skeletal from dentoalveolar effects, and experimental studies that investigate cellular or molecular events during maxillary expansion. Orthodontic tooth movement and broader bone mechanobiology are used only as supporting biological contexts when direct expansion-specific evidence is unavailable. Throughout the manuscript, “demonstrates” denotes a direct observation in the cited model, “supports” denotes convergent but indirect evidence, and “we propose” marks a hypothesis that requires validation.

## 2. Current Evidence Across Tissue Domains

### 2.1. Midpalatal and Circummaxillary Sutural Response

The midpalatal suture is the most directly visualized skeletal site of maxillary expansion, but it does not function in mechanical isolation. Expansion forces are transmitted through the maxillary complex and surrounding articulations, and resistance at posterior and circummaxillary structures may influence both the magnitude and the spatial pattern of midpalatal separation. Mechanical and imaging studies have demonstrated that sutural opening may be parallel, anteriorly dominant, or otherwise heterogeneous, depending on appliance configuration, skeletal maturity, local resistance, and the effectiveness of force transfer [7,8,20,21].

Evidence that the suture undergoes active biological remodeling is derived primarily from experimental models and a limited number of human observations. Cellular changes, osteoclast-related signaling, angiogenic mediator release, immune-cell participation, and osteogenic activity have all been reported within the expanded sutural environment [9,10,11,12].

Human clinical evidence is stronger for structural than for molecular outcomes. CBCT and other three-dimensional approaches can document sutural separation, basal displacement, anterior–posterior opening patterns, and progressive radiographic mineral recovery during retention. These findings support the concept of an active skeletal response but do not directly identify the cellular mechanism responsible for the observed image changes [16,22].

Human histological and imaging evidence further indicates that midpalatal suture morphology and interdigitation change with maturation, although chronological age is an imperfect surrogate for individual sutural status. Histological observations demonstrate substantial age-related variability in palatal growth and suture closure, whereas CBCT-based maturation assessment and its systematic evaluation support individualized rather than age-only interpretation before expansion [23,24,25,26].

A 2025 systematic review and meta-analysis of 17 studies documented widening of the midpalatal suture and nasal cavity in late adolescents and young adults. Greater dental side effects were associated with smaller sutural opening, indicating that similar transverse corrections may contain different skeletal and dentoalveolar components [27]. In a separate CBCT cohort of 82 patients, pterygomaxillary splitting occurred in approximately half of the sample and increased with posterior palatal expansion. It was less pronounced in older patients and in those with greater sutural maturation or higher ANB values [28]. A 2026 CBCT study likewise showed that chronological age functions only as a decision-support marker. Individual assessment of sutural maturation remains necessary for expansion planning [29].

### 2.2. Alveolar and Dentoalveolar Response

Alveolar bone is a mechanosensitive tissue that deforms and remodels under expansion-related strain [30]. Clinical expansion may therefore include varying combinations of alveolar bending, dental tipping, translation, and cortical remodeling. Although these components may increase arch width, they are not biologically equivalent to sutural or basal skeletal displacement. Earlier computed tomography evidence showed that dental and alveolar widening may exceed basal skeletal widening, reinforcing the need to distinguish total arch expansion from orthopedic correction [31].

Three-dimensional clinical studies demonstrate that appliance design and anchorage configuration influence the relative distribution of skeletal, alveolar, and dental effects. Tooth-borne systems generally transmit a greater proportion of load through the dentition and periodontal support, whereas bone-anchored or tooth–bone-borne systems may increase the skeletal contribution. However, neither system eliminates dentoalveolar response, and substantial inter-individual variability remains [1,2,3,18]. Systematic and scoping reviews likewise report substantial variation in MARPE efficacy and in the balance of skeletal and dentoalveolar effects across populations, appliances, outcome definitions, and study designs [32,33,34].

Alveolar response should not be classified automatically as either a beneficial adaptation or pathological damage. Limited bending and remodeling may accommodate the altered transverse geometry. Marked dental inclination, buccal cortical loss, or root displacement beyond the alveolar envelope indicates greater tissue burden. Interpretation depends on the spatial distribution of change, baseline anatomy, periodontal phenotype, and subsequent stability. In a 2025 randomized trial, hybrid MARPE produced greater nasal, basal maxillary, and alveolar widening and less buccal bone thinning than tooth-borne RPE, although posterior dental inclination increased with both appliances [35].

### 2.3. Anchorage-Related Periodontal and Periodontal Ligament Response

Orthodontic tooth movement helps explain the response of anchorage teeth, although it does not model sutural biology. Mechanical loading of an erupted tooth produces spatially heterogeneous compression and tension within the periodontal ligament and adjacent alveolar bone, accompanied by vascular alteration, inflammatory mediator release, osteoclast recruitment, and matrix turnover [36,37].

During maxillary expansion, the periodontal ligament is loaded through anchorage teeth and through dentoalveolar deformation. Gingival crevicular fluid studies have documented changes in IL-1β, TGF-β1, PGE_2_, and nitric oxide around anchorage teeth during active expansion and retention, confirming that the periodontal tissues participate in the biological response [19]. However, gingival crevicular fluid is sampled from the periodontal environment. Its molecular profile should therefore be interpreted as an anchorage-related periodontal readout and not as a surrogate measurement of cellular activity within the midpalatal suture.

Additional expansion-specific studies have evaluated IL-1β, oxidative status, and broader stress-response indicators during rapid maxillary expansion. These measurements are best treated as periodontal or stress-response readouts, not as markers of sutural bone formation [38,39].

Periodontal effects reported after expansion include changes in buccal bone support, gingival recession, root resorption, and other anchorage-related outcomes, although their frequency and clinical importance are heterogeneous across studies [17]. The PDL is clinically relevant because it determines whether dentally transmitted forces remain within the adaptive capacity of the supporting tissues. A 2026 systematic review of 20 clinical studies reported frequent dental tipping, alveolar bone loss in 11 studies, buccal dehiscence in three, and failure of sutural opening in six. However, the overall certainty of evidence was judged to be low to very low because of methodological heterogeneity, risk of bias, and limited or absent follow-up in many studies [40].

### 2.4. The Unresolved Integration Problem

No single outcome captures the biology of maxillary expansion. Sutural opening does not quantify dentoalveolar burden, arch widening does not identify the skeletal component, and periodontal biomarkers do not describe sutural remodeling. The central question is how the applied load is distributed among these domains and why that distribution differs between patients. This question motivates the three-compartment model.

## 3. Mechanistic Interpretation of Sutural Remodeling

### 3.1. Early Mechanosensing and Margin Turnover

Expansion produces deformation of the sutural connective tissue, displacement of the bony margins, and altered strain within the surrounding skeletal structures. Experimental evidence indicates that this mechanical environment is followed by activation of cellular programs involved in tissue turnover and osteogenesis. RANK/RANKL/OPG expression has been demonstrated during rapid maxillary expansion, supporting participation of osteoclast-regulatory signaling in remodeling of the sutural margins [9].

More recent studies identify several mechanosensitive and osteogenic pathways within experimental expansion models. FTO has been associated with osteogenic differentiation of suture mesenchymal stem cells, Piezo1 signaling has been linked to CaMKII-dependent osteogenesis, and mechanical tension has been shown to activate a Dalrd3–Id3 translational mechanism in bone suture stem cells [13,14,41]. Earlier experimental work also implicated peroxisome proliferator-activated receptor-γ in bone remodeling after midpalatal suture expansion in mice, providing further evidence that sutural remodeling involves multiple, partly distinct regulatory pathways rather than a single universal molecular cascade [42].

Together, these studies demonstrate mechanosensitive signaling in experimental sutural models, but they do not define a single pathway because the species, loading protocols, and observation windows differ. Several tension-responsive mechanisms are plausible; their relative importance and hierarchy remain unknown.

### 3.2. Immune and Vascular Coupling

Immune-cell recruitment and vascular adaptation also occur within the expanded sutural environment. Neutrophils have been implicated in early bone formation during midpalatal expansion, whereas mechanically induced M2 macrophage phenotypes have been associated with subsequent bone remodeling [11,12]. These findings indicate that immune cells may participate in the regulation of tissue turnover rather than representing only nonspecific inflammation.

VEGF and FGF-2 release has also been demonstrated within the palatal suture after rapid expansion, supporting angiogenic involvement in the remodeling environment [10]. Vascular adaptation is biologically relevant because tissue expansion, matrix turnover, and new bone formation require maintenance or restoration of an adequate microvascular supply.

The current evidence does not establish whether immune-cell recruitment, vascular change, or direct mechanosensing acts as a single initiating event. A more evidence-consistent interpretation is that these processes interact and overlap. Hypoxia-sensitive signaling may contribute under selected local conditions, but direct evidence is insufficient to identify hypoxia as a universal upstream regulator of human maxillary expansion. Accordingly, hypoxia should be presented as a possible context-dependent modulator rather than as the principal explanatory mechanism.

### 3.3. Osteogenesis, Mineralization, and Stabilization

Following mechanical separation, the expanded sutural region must regain structural continuity if the skeletal correction is to stabilize. Experimental studies demonstrate osteogenic differentiation and new bone formation within expanded sutures, whereas clinical imaging studies show progressive radiographic recovery of mineralized tissue during retention [13,15,16,41].

Radiographic bone fill does not demonstrate complete histological restoration. CBCT-derived density or visual bone-fill scores characterize mineralized tissue at the imaging scale but do not directly establish cellular composition, matrix maturity, vascular organization, or mechanical competence. The term “progressive mineralization and restoration of structural continuity” is therefore more accurate than an unqualified claim of complete re-ossification.

### 3.4. Proposed Temporal Integration and Evidentiary Limits

The available studies suggest overlapping phases of sutural remodeling. An immediate mechanical phase is followed by cellular recruitment and margin turnover, while immune, vascular, and osteogenic processes develop across partially overlapping intervals. During stabilization and retention, matrix deposition and progressive mineralization contribute to restoration of structural continuity.

**Figure 1:**
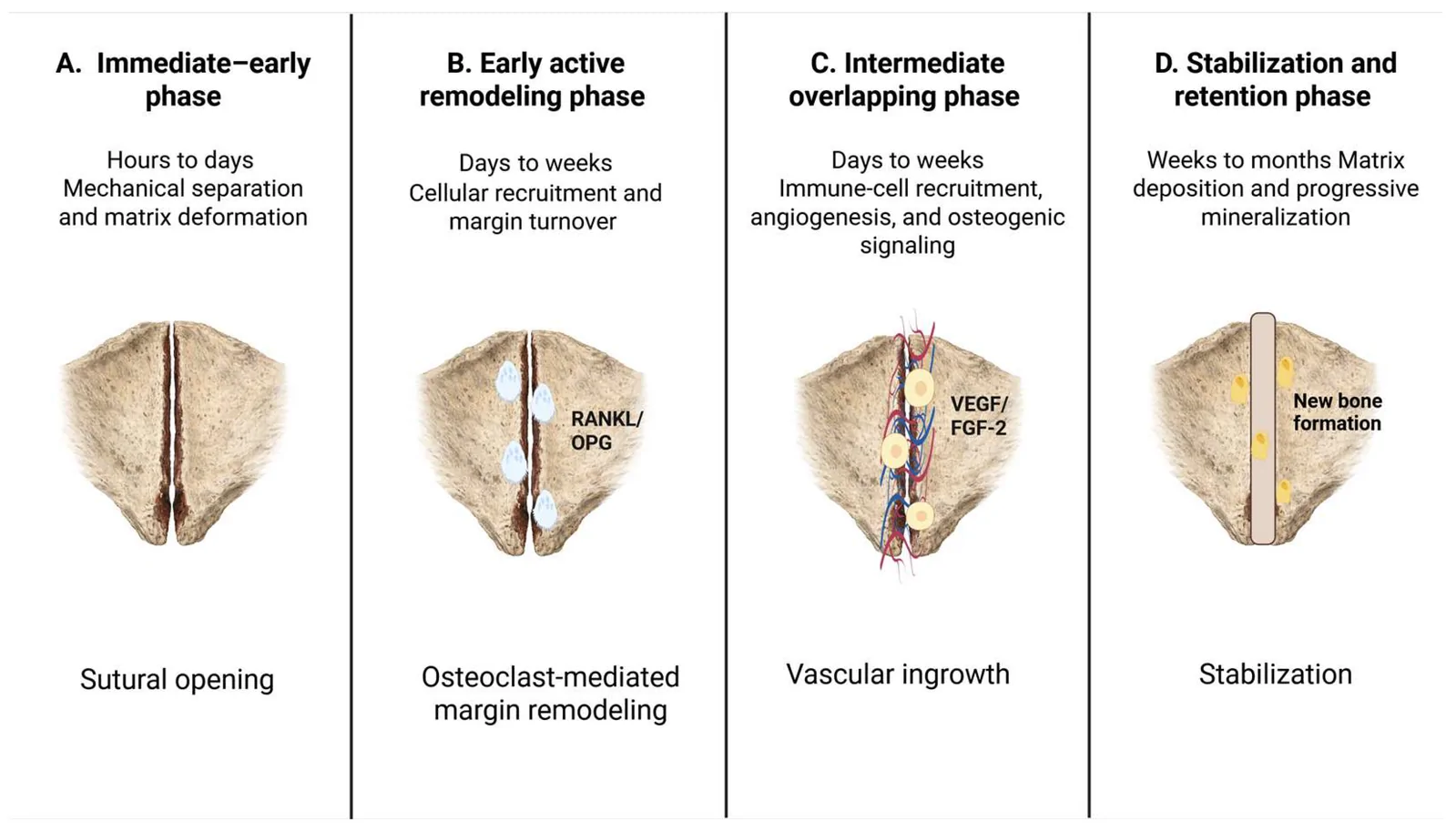
Proposed temporal organization of sutural remodeling during maxillary expansion. Mechanical separation produces an immediate change in sutural geometry and matrix strain. During the early active remodeling phase, cellular recruitment and osteoclast-related turnover occur at or near the sutural margins. Immune-cell participation, vascular and angiogenic adaptation, and osteogenic signaling develop across partially overlapping intervals. During stabilization and retention, osteoblastic matrix deposition and progressive mineralization contribute to restoration of structural continuity. The indicated time ranges are schematic rather than fixed clinical intervals. The phases may overlap, and their duration varies with species, age, sutural maturity, appliance design, force magnitude, activation rhythm, and retention conditions. The panel sequence represents a proposed temporal integration of available findings and not a validated linear causal cascade. Colored elements are schematic: blue elements indicate remodeling-related cellular activity, red structures indicate vascular ingrowth, and yellow elements indicate osteogenic activity or new bone deposition. RANKL, receptor activator of nuclear factor κB ligand; OPG, osteoprotegerin; VEGF, vascular endothelial growth factor; FGF-2, fibroblast growth factor 2.

The onset, duration, and overlap of these processes vary with species, age, sutural maturity, force magnitude, activation rhythm, appliance design, and retention conditions. Figure 1 summarizes the proposed sequence schematically and does not establish a linear causal cascade.

## 4. Proposed Evidence-Informed Three-Compartment Framework

### 4.1. Definitions and Conceptual Boundaries

We organize these findings into three analytic compartments. Here, “compartment” denotes a measurement domain, not an anatomically isolated system. Mechanical forces, vascular networks, cellular signals, and structural adaptations interact across domain boundaries.

The sutural skeletal domain comprises the midpalatal suture and the surrounding sutural and skeletal structures that participate in orthopedic displacement. It is designated as the principal intended orthopedic target because skeletal correction is the defining therapeutic objective of maxillary expansion.

The alveolar and dentoalveolar domain is defined as an adaptive load-transfer domain. It includes alveolar bending and remodeling, dental inclination, translation, and changes in cortical support. These responses may contribute to correction, compensate for limited sutural displacement, or generate non-target tissue burden.

The anchorage-related periodontal domain includes periodontal ligament deformation, local inflammatory and vascular responses, root-related effects, and gingival or crestal consequences around anchorage teeth. It is designated as a non-target response domain because periodontal loading is not the principal therapeutic objective, although its magnitude may determine the biological acceptability of treatment.

**Figure 2:**
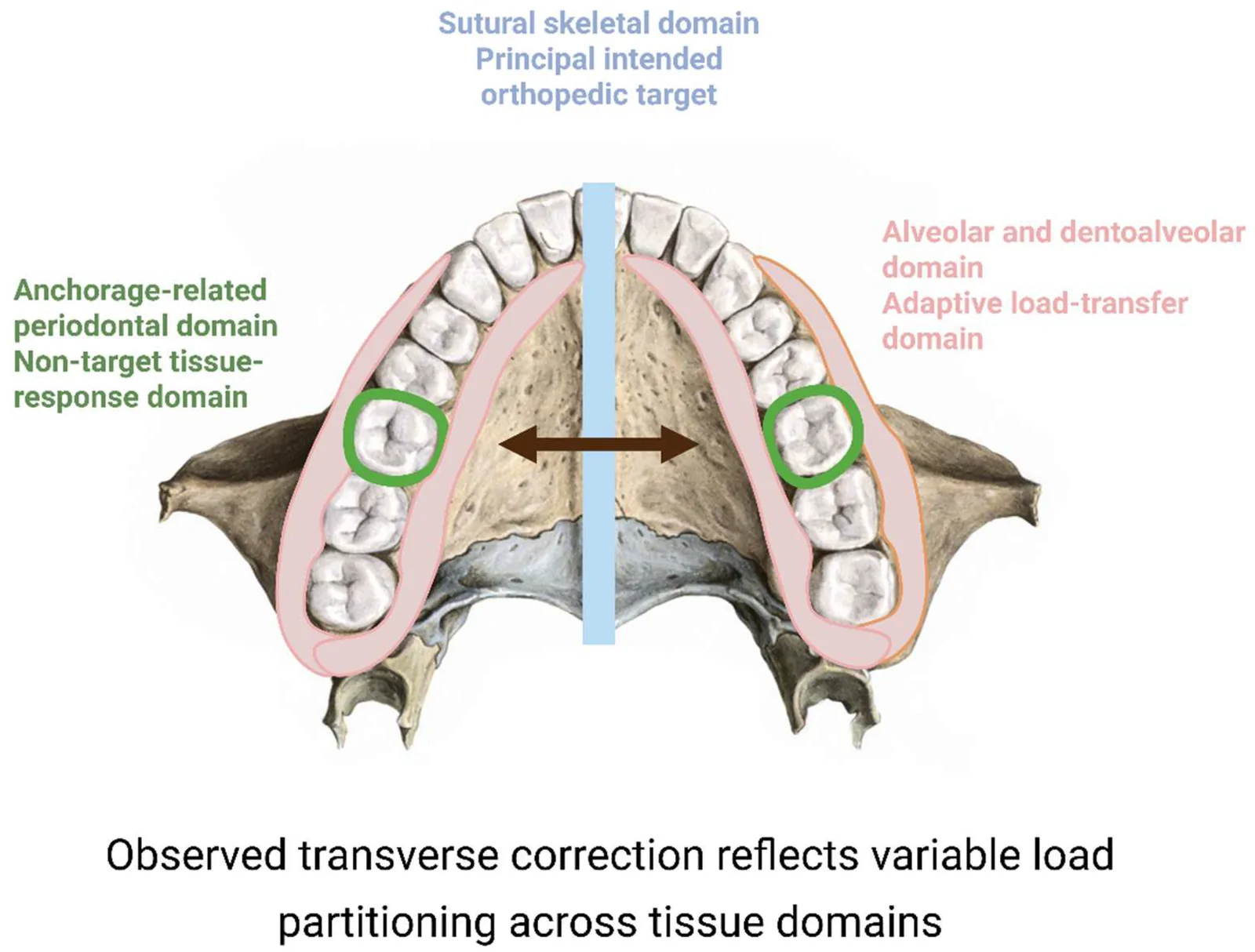
Three-compartment model of maxillary expansion. The sutural skeletal domain comprises the intended orthopedic target and the associated circummaxillary skeletal response. The alveolar and dentoalveolar domain includes bending, remodeling, dental inclination, and translation. The anchorage-related periodontal domain includes non-target PDL and periodontal effects. The domains interact and may respond concurrently; their labels indicate treatment roles, not isolated anatomy, fixed timing, or validated patient-level thresholds. The bidirectional arrow indicates transverse expansion and load transfer across the midpalatal region, whereas the green circles identify the anchorage-related periodontal domains around the posterior teeth.

The terms principal, adaptive, and non-target do not specify a temporal order. Alveolar and periodontal responses may begin concurrently with sutural loading and may account for a substantial proportion of the observed transverse correction. Similarly, the designation of the suture as the principal intended target does not imply that sutural remodeling is biologically dominant in every patient. The proposed roles of the three tissue domains and their contribution to load partitioning during maxillary expansion are summarized in Figure 2.

### 4.2. Operational Readouts

Estimating the contribution of each domain requires combined structural, dental, and periodontal measures. Suggested sutural measures include anterior and posterior midpalatal opening, basal maxillary displacement, nasal-floor or maxillary-width change, circummaxillary sutural response, and mineralized tissue recovery. Alveolar and dentoalveolar measures include alveolar bending, dental inclination and translation, cortical thickness, and root position within the alveolar envelope. Periodontal measures include gingival recession, inflammatory findings, root resorption, crestal support, and, in research settings, gingival crevicular fluid biomarkers.

These measurements do not currently provide validated thresholds for assigning a patient to a single compartment. They should instead be used to estimate the relative tissue contribution to the observed outcome. The same patient may exhibit substantial responses in all three domains.

### 4.3. Testable Predictions

The framework generates several falsifiable predictions. First, among patients achieving similar total transverse correction, a greater sutural and circummaxillary skeletal contribution should be associated with a lower relative proportion of dental tipping and alveolar bending. Second, patient-specific mechanical estimates of high strain within the alveolar cortex or periodontal ligament should predict greater dentoalveolar or periodontal burden independently of the nominal appliance activation. Third, periodontal biomarkers obtained from gingival crevicular fluid should correlate more strongly with anchorage-related tissue response than with subsequent sutural mineralization. Fourth, tissue-specific molecular analysis should demonstrate partially distinct temporal and cellular profiles in the suture, alveolar bone, and periodontal ligament rather than a single uniform inflammatory response across all tissues (see Table 1).

The distinction between “established observations” and “proposed interpretive role” is intentional. The framework organizes available evidence but does not assume that the three domains are anatomically isolated, temporally sequential, or quantitatively validated.

## 5. Clinical Interpretation

This model is an interpretive tool, not a treatment-selection algorithm. It does not establish an evidence-based threshold for choosing tooth-borne expansion, MARPE, or surgically assisted expansion, and it should not be used to infer individual safety without appropriate clinical and radiographic assessment.

Clinically, arch-width increase alone is insufficient. Outcome reporting should distinguish the sutural and circummaxillary skeletal component from alveolar deformation, dental movement, and periodontal consequences. Two patients with similar intermolar widening may therefore have biologically different outcomes if one exhibits predominantly skeletal displacement and the other shows a larger contribution from alveolar bending and dental tipping.

Tooth-borne, tooth–bone-borne, and bone-anchored systems distribute forces differently. Their effects are further modified by skeletal maturity, circummaxillary resistance, palatal anatomy, bone quality, periodontal support, miniscrew position, appliance rigidity, and activation protocol [3,18,21].

Stability should also be evaluated by tissue domain. Skeletal retention, dental relapse, alveolar adaptation, and periodontal health are related but non-equivalent outcomes. Prospective evidence shows that skeletal and dental components may exhibit different relapse trajectories after MARPE, reinforcing the need to avoid treating total width as a biologically uniform endpoint [43,44]. These recent findings extend earlier longitudinal evidence that dental, alveolar, and skeletal components do not necessarily remain stable to the same degree after expansion and that relapse should be reported by tissue domain rather than as a single transverse measure [45,46]. Recent evidence further suggests that pretreatment skeletal subtype modifies three-dimensional maxillary displacement and secondary mandibular rotation after MARPE, reinforcing that appliance category alone is insufficient to predict the individual tissue-level response [47].

## 6. Validation Agenda for the Three-Compartment Framework

The proposed three-compartment framework is intended to be empirically testable rather than solely descriptive. Its validity cannot be inferred from conceptual coherence alone. Validation requires evidence that the sutural skeletal, alveolar and dentoalveolar, and anchorage-related periodontal domains can be measured separately, display distinguishable tissue-level response patterns, and improve prediction of clinical outcomes beyond conventional variables such as chronological age, appliance category, or nominal activation protocol.

The predictions presented in Section 4.3 can be examined through three complementary research strategies: prospective multimodal clinical assessment, compartment-resolved experimental studies, and patient-specific computational–clinical validation. These approaches address different levels of evidence. Clinical studies can determine whether the proposed domains correspond to reproducible patient-level outcomes. Experimental models can evaluate whether the domains display distinct cellular and molecular responses. Computational models can test whether the pretreatment distribution of mechanical loading predicts the subsequent tissue-specific outcome.

### 6.1. Prospective Multimodal Clinical Validation

A prospective multicenter cohort should enroll consecutive patients with transverse maxillary deficiency treated using prespecified tooth-borne, tooth–bone-borne, or bone-anchored expansion protocols. Appliance categories should not be pooled as a single exposure. Appliance rigidity, anchorage configuration, activation magnitude and rhythm, miniscrew number and position, palatal morphology, skeletal pattern, sutural maturation, baseline alveolar anatomy, and periodontal phenotype should be documented prospectively as potential determinants of load partitioning.

Baseline and post-expansion low-dose CBCT examinations, when clinically justified, should be combined with serial intraoral scans and standardized periodontal assessment. Repeated radiographic exposure should be minimized. Digital models and clinical periodontal measurements can therefore provide non-ionizing longitudinal follow-up during stabilization and retention. Existing prospective and three-dimensional studies demonstrate that sutural opening, basal maxillary displacement, alveolar inclination, dental tipping, and cortical changes can be measured separately rather than treated as one pooled transverse outcome. They also demonstrate substantial variation in the relative skeletal and dentoalveolar components of expansion among patients treated with broadly comparable protocols [1,2,3,18].

The primary endpoint should not be total intermolar or interpremolar width increase. Outcomes should instead be prespecified within the three proposed domains. Sutural skeletal outcomes should include anterior and posterior midpalatal opening, basal maxillary displacement, nasal-floor or maxillary-width change, circummaxillary sutural response, and progressive restoration of mineralized tissue during retention. Alveolar and dentoalveolar outcomes should include alveolar bending, dental inclination and translation, buccal and palatal cortical thickness, and root position relative to the alveolar envelope. Periodontal outcomes should include gingival recession, bleeding on probing, probing depth, crestal support, root resorption, and anchorage-related tissue complications.

Gingival crevicular fluid may be sampled around anchorage teeth to evaluate local periodontal responses. However, these biomarkers should be assigned prospectively to the anchorage-related periodontal domain. They should not be interpreted as surrogate markers of cellular activity within the midpalatal suture. Temporal changes in IL-1β, TGF-β1, PGE_2_, and nitric oxide during rapid maxillary expansion support the feasibility of monitoring periodontal biological activity, but not of inferring sutural remodeling from periodontal fluid samples [19].

The analysis should quantify the relative contribution of each tissue domain to the total observed correction. Multilevel models should account for repeated measurements, clustering within treatment centers, and differences in appliance configuration. Total transverse correction should be included as a covariate rather than treated as the principal biological endpoint. A compositional or latent-variable approach could subsequently be used to derive a compartmental profile, but only after the reproducibility and independence of the individual measurements have been established.

Support would require an inverse association between sutural skeletal contribution and dentoalveolar compensation after adjustment for relevant covariates. Periodontal outcomes should correlate preferentially with anchorage-related loading rather than with later sutural mineralization. Failure to distinguish domain-specific outcomes, or equivalent prediction by total transverse width alone, would argue against the model.

At least one retention-phase assessment should be included because the skeletal, dental, and alveolar components of expansion may display different stability trajectories. Short-term correction and longer-term biological stability should therefore be evaluated as related but non-equivalent outcomes [43,44,45].

### 6.2. Compartment-Resolved Experimental Validation

A controlled experimental study should evaluate the midpalatal suture, alveolar bone, and periodontal ligament separately within the same expansion model. Assessing all three domains in the same animals would reduce confounding caused by differences in species, loading protocol, force magnitude, and observation period. The intervention should use a standardized expansion force and activation schedule, with tissue collection at species-appropriate early, active-remodeling, and stabilization time points.

The sutural skeletal domain should be analyzed using micro-computed tomography, histomorphometry, fluorochrome labeling where appropriate, osteoclast and osteoblast markers, vascular imaging, and compartment-specific molecular analysis. The alveolar domain should be evaluated for cortical deformation, microdamage, remodeling activity, osteocyte response, and changes in buccal and palatal bone architecture. The periodontal ligament should be examined separately for compression- and tension-related deformation, vascular disturbance, inflammatory mediator expression, root-surface effects, and adjacent alveolar remodeling.

Candidate sutural readouts may include RANK/RANKL/OPG signaling, osteoclast activity, osteoblast-lineage differentiation, angiogenic mediators, neutrophil recruitment, macrophage phenotypes, and mechanosensitive signaling pathways. Existing experimental studies provide evidence for RANK/RANKL/OPG involvement, VEGF and FGF-2 release, neutrophil participation, mechanically induced M2 macrophage activity, and Piezo1-mediated osteogenic signaling during palatal expansion [9,10,11,12,13].

More recent studies additionally identify tension-responsive osteogenic mechanisms involving FTO and the Dalrd3–Id3 axis in suture mesenchymal or bone suture stem-cell populations. These pathways may be incorporated as candidate mechanosensitive readouts, but they should not be assumed to represent one obligatory molecular sequence [14,41].

Where technically feasible, spatial transcriptomic analysis, single-cell profiling, or targeted compartment-specific transcript panels could be used to determine whether the same expansion stimulus produces different dominant cellular programs in the suture, alveolar bone, and periodontal ligament. Spatially resolved analysis is important because homogenizing all maxillary tissues into one sample would obscure the tissue specificity that the framework is intended to test.

The framework would be supported if the three tissue domains demonstrated reproducibly different spatial and temporal response profiles. For example, the suture would be expected to show direct mechanosensing, margin turnover, angiogenic activity, and osteogenic consolidation; alveolar bone would be expected to demonstrate load-dependent deformation and remodeling; and the periodontal ligament would be expected to show an anchorage-related inflammatory and vascular response. The framework would require refinement if the same molecular profile occurred uniformly across all tissues, if the proposed domains could not be distinguished analytically, or if periodontal responses more accurately predicted sutural bone formation than direct sutural measurements.

The experimental design should also test temporal overlap rather than assume a fixed linear cascade. Early immune-cell recruitment, vascular adaptation, osteoclast-related turnover, and osteogenic signaling may occur concurrently. The relevant question is therefore not whether one event always precedes another, but whether their relative magnitude and localization differ among tissue domains and across phases of treatment.

### 6.3. Patient-Specific Computational–Clinical Validation

Patient-specific finite element modeling offers a complementary method for testing whether pretreatment mechanical conditions predict the subsequent distribution of tissue response. Models should be constructed from pretreatment three-dimensional anatomy and should incorporate the midpalatal and relevant circummaxillary sutures, cortical and trabecular bone, teeth, periodontal ligament, appliance components, and miniscrews where applicable. Palatal cortical thickness, miniscrew insertion position, appliance geometry, and the relationship between the expansion screw and the center of resistance should be represented explicitly.

The model should generate separate mechanical outputs for the three domains. Sutural outputs may include opening tendency, principal strain, strain-energy density, and spatial gradients along the anterior and posterior suture. Alveolar outputs may include buccal and palatal cortical strain, bending patterns, and local stress concentrations. Periodontal outputs may include compression, tension, hydrostatic stress, or other mechanically appropriate measures within the periodontal ligament around anchorage teeth.

Material properties should not be treated as perfectly known constants. Sensitivity analyses should evaluate how variation in sutural stiffness, cortical density, trabecular properties, periodontal ligament behavior, and miniscrew–bone coupling affects predicted load partitioning. This is necessary because otherwise a visually precise model may provide falsely precise biological predictions.

Previous mechanical and biomechanical studies demonstrate that expansion forces are distributed through the broader facial skeleton and that appliance design and anchorage configuration influence the resulting stress and displacement patterns [20,21].

Clinical studies further show that similar appliances may produce heterogeneous skeletal and dentoalveolar opening patterns, providing an appropriate outcome set for prospective computational validation [2,7].

Predicted compartmental loading should be compared prospectively with observed outcomes. Higher modeled sutural strain should be tested against the magnitude and pattern of sutural opening and basal maxillary displacement. Higher alveolar cortical strain should be tested against subsequent alveolar bending, cortical thickness changes, and dental inclination. Higher periodontal ligament loading around anchorage teeth should be tested against dental tipping, periodontal inflammatory findings, root-related outcomes, and gingival changes.

The framework would be supported if pretreatment mechanical estimates predicted the corresponding tissue-specific outcomes and improved prediction beyond chronological age, sutural maturation, appliance category, and nominal activation alone. Model calibration should be performed in one cohort and evaluated through internal cross-validation. External validation should then be undertaken in an independent sample. The framework would be weakened if predicted sutural, alveolar, and periodontal loading showed no preferential association with their corresponding clinical outcomes or if a simpler non-compartmental model performed equally well.

### 6.4. Criteria for Confirmation, Refinement, or Rejection

No single study can validate the complete framework. Confirmation requires convergent evidence across structural, biological, and computational levels. Four forms of validity should therefore be considered.

First, construct validity requires that the proposed domains can be represented by reproducible and clinically interpretable measurements. Sutural opening, alveolar bending, dental inclination, and periodontal findings should not collapse into one undifferentiated transverse outcome.

Second, discriminant validity requires that the selected readouts show stronger associations with their proposed tissue domain than with the other domains. Periodontal biomarkers should preferentially reflect anchorage-related periodontal loading. Sutural molecular and imaging measures should preferentially characterize the skeletal response. Alveolar cortical changes should correspond to the dentoalveolar load-transfer pathway.

Third, predictive validity requires that pretreatment anatomical, biological, or mechanical variables predict the subsequent distribution of skeletal, alveolar, and periodontal outcomes. A compartmental model should add information beyond established predictors such as age, appliance design, and sutural maturation.

Fourth, external validity requires replication across different centers, patient populations, appliance systems, and activation protocols. Results derived from one appliance or one age group should not be assumed to generalize automatically to all forms of maxillary expansion.

The framework should be refined if one proposed domain consistently separates into biologically distinct subdomains, if relevant tissue interactions are not represented adequately, or if the terminology does not correspond to measurable outcomes. It should be rejected in its current form if the proposed compartments cannot be distinguished reproducibly, if their measurements provide no explanatory or predictive advantage over total transverse change, or if tissue-specific biological findings consistently contradict the proposed allocation of response domains.

Until these criteria are met, the framework should remain a provisional analytic model. It should not be used to assign exact patient-specific percentages of skeletal gain or dentoalveolar burden, to define universal safety thresholds, or to prescribe a specific appliance solely on the basis of the three-compartment classification. Its immediate value lies in generating testable hypotheses, standardizing tissue-specific outcome reporting, and providing a structured pathway from mechanobiological interpretation to prospective validation.

## 7. Limitations of the Framework

Direct human molecular data are sparse because repeated sampling of the midpalatal suture is rarely feasible. Mechanistic interpretation therefore relies heavily on animal and in vitro models, which differ from humans in sutural anatomy, maturation, loading, and healing.

CBCT can separate skeletal, alveolar, and dental changes, but it cannot identify their molecular basis. Radiographic bone fill does not establish histological maturity or mechanical competence. The three compartments are also biologically interconnected, so the model should not be read as a map of isolated tissue systems.

Clinical studies vary in appliance design, activation protocol, imaging schedule, patient age, skeletal maturity, and outcome definitions. No validated thresholds define when alveolar or periodontal adaptation becomes disproportionate or clinically unacceptable. The model remains organizational and hypothesis-generating pending prospective validation.

## 8. Conclusions

Maxillary expansion combines sutural skeletal displacement with variable alveolar, dental, and periodontal responses. Comparable increases in arch width can therefore arise from different tissue contributions.

Experimental studies demonstrate active mechanosensing, immune and vascular participation, and osteogenesis within expanded sutures, but the sequence and relative importance of these processes in humans remain uncertain.

We propose three analytic domains—sutural skeletal, alveolar and dentoalveolar, and anchorage-related periodontal—to report these responses separately. Prospective imaging, biological sampling, histology, and patient-specific modeling are needed to determine whether this distinction improves prediction of treatment response and tissue burden.

## Figures and Tables

**Table 1:** Evidence, proposed interpretive roles, operational readouts, and testable predictions across tissue domains engaged during maxillary expansion.

Tissue Domain	Established Observations	Proposed Interpretive Role	Candidate Operational Readouts	Testable Prediction
Sutural skeletal domain	Midpalatal separation and heterogeneous opening patterns are documented clinically. Experimental studies demonstrate cellular turnover, immune and vascular participation, and osteogenic signaling within expanded sutures. Direct longitudinal human molecular evidence remains limited [2,3,8,10,11,12,13,18,27,28].	Principal intended orthopedic target contributing to sutural and circummaxillary skeletal correction.	Anterior and posterior sutural opening; basal maxillary and nasal-floor displacement; circummaxillary sutural change; progressive mineralized tissue recovery during retention.	For comparable total transverse correction, a greater sutural skeletal contribution should be associated with a lower relative proportion of dental tipping and alveolar bending.
Alveolar and dentoalveolar domain	Alveolar inclination, bending, dental tipping, changes in buccal cortical thickness, and appliance-dependent differences have been documented by three-dimensional clinical studies [1,2,3,18,27,35,40].	Adaptive load-transfer domain that may support correction or compensate for limited sutural displacement.	Alveolar inclination; dental tipping and translation; buccal and palatal cortical thickness; root position within the alveolar envelope.	Greater modeled or measured alveolar loading should predict greater dentoalveolar compensation independently of total arch-width increase.
Anchorage-related periodontal domain	Periodontal inflammatory mediator changes, periodontal loading, and heterogeneous periodontal side effects have been reported around anchorage teeth [1,17,19,40].	Non-target tissue-response domain reflecting the biological consequences of force transmission through the dentition.	Gingival recession; bleeding and probing measures; root resorption; periodontal support; GCF biomarkers in research settings.	Periodontal biomarkers and adverse outcomes should correlate more strongly with anchorage-related loading than with the magnitude of sutural bone formation.

## Data Availability

No new data were created or analyzed in this study.

## Abbreviations

RPE	Rapid Palatal Expansion
MARPE	Miniscrew-Assisted Rapid Palatal Expansion
PDL	Periodontal Ligament
RANK	Receptor Activator of Nuclear Factor κB
RANKL	Receptor Activator of Nuclear Factor κB Ligand
OPG	Osteoprotegerin
